# Identification of Hub genes associated with infection of three lung cell lines by SARS‐CoV‐2 with integrated bioinformatics analysis

**DOI:** 10.1111/jcmm.15862

**Published:** 2020-09-14

**Authors:** Tian‐Ao Xie, Meng‐Yi Han, Xiao‐Rui Su, Hou‐He Li, Ji‐Chun Chen, Xu‐Guang Guo

**Affiliations:** ^1^ Department of Clinical Medicine The Third Clinical School of Guangzhou Medical University Guangzhou China; ^2^ Department of Clinical Laboratory Medicine The Third Affiliated Hospital of Guangzhou Medical University Guangzhou China; ^3^ Key Laboratory for Major Obstetric Diseases of Guangdong Province The Third Affiliated Hospital of Guangzhou Medical University Guangzhou China; ^4^ Key Laboratory of Reproduction and Genetics of Guangdong Higher Education Institutes The Third Affiliated Hospital of Guangzhou Medical University Guangzhou China

## 1 INTRODUCTION

In December 2019, 41 cases of pneumonia of unknown aetiology broke out in Wuhan city, Hubei Province, China.^1^ Later on, officially named as SARS‐CoV‐2 by the Coronavirus Study Group of the International Committee on Taxonomy of Viruses after it is recognized as a sister virus of the prototype human and bat severe acute respiratory syndrome coronaviruses (SARS‐CoVs).^2^

Coronaviruses are a group of viruses that induce infections of respiratory tract and intestines in animals and humans, including four types: α, β, γ and δ.^3^ SARS‐CoV‐2, as a positive‐sense single‐stranded RNA β‐coronavirus. SARS‐CoV‐2 shares sequence homology with Middle East Respiratory Syndrome Coronavirus (MERS‐CoV; 50% homology) and Severe Acute Respiratory Syndrome Coronavirus (SARS‐Cov‐1; 79% homology).^1^

SARS‐CoV‐2 is thought to be transmitted mainly through close contacts between people, respiratory droplets or aerosols carrying viruses.^4^ Up to 22 May 2020, it has spread to over 216 countries over the world, with 4 995 996 confirmed cases, including 327 821 deaths.^5^

At present, there are no effective drugs available for the treatment of COVID‐19. The genetic diversity and frequent recombination of coronavirus genomes render the variation of coronaviruses highly unpredictable. Therefore, exploring biomarkers of SARS‐CoV‐2 with a combination of integrated bioinformatics methods with expression profiling techniques is hopefully helpful for improving the diagnosis, treatment and prognosis of SARS‐CoV‐2 in the future.

This study focused on gene expression in three types of cells infected with SARS‐CoV‐2, including primary human lung epithelium (NHBE), transformed lung alveolar (A549) cells and transformed lung‐derived Calu‐3 cells. The original microarray data of GSE147507 were obtained from Gene Expression Omnibus (GEO). The study was designed to identify key biomarker candidates for SARS‐CoV‐2 and improve the diagnosis and prognosis based on functional and molecular analyses by evaluating DEGs in three groups.

## 2 METHODS AND MATERIALS

### 2.1 Data inclusion and DEG screening

The gene expression profile of GSE147507 (https://www.ncbi.nlm.nih.gov/geo/query/acc.cgi?acc=GSE147507) in this study was obtained from National Center for Biotechnology Information Gene Expression Omnibus (http://www.ncbi.nlm.nih.gov/geo/), on the basis of GPL18573 platform of Illumina NextSeq 500 (*Homo sapiens*) and GPL28369 platform of Illumina NextSeq 500 (*Mustela putorius furo)*. In the original study, the researchers set up a human group and a ferret group and performed the experiment by transfecting NBHE, A549, lung‐derived Calu‐3 cells in human groups with SARS‐CoV‐2 and Influenza A virus (IAV), the latter lacking the NS1 protein (IAVdNS1) in triplicate data. These data can be obtained from GPL18573 platform. For the purpose of studying SARS‐CoV‐2, only data from the human group were extracted for research, specifically data of human lung proto‐epithelium (NHBE; GSM4432378‐83, GSM4462363‐66), alveolar cells in GSE147507 (A549; GSM4432384‐91, GSM4432394‐95, GSM4462336‐47, GSM4462354‐56 and GSM4486157‐62) and transformed lung‐derived Calu‐3 cells (GSM4462348‐53). The GSE147507 series of matrix file data sets were downloaded, the gene probes were converted into gene names on the GPL18573 platform, and the matrix of data counts was convert to tpm format. Then, the limma software package in R software was used to standardize and screen each set of data, with the screening criteria set as: |log2FC| > 1 and *P* < .01 for the purpose of identifying genes with significant changes.

### 2.2 Functional and pathway enrichment analyses of DEGs

To identify the biological function of DEGs, this study employed the Gene Ontology (GO) and Kyoto Encyclopedia of Genes and Genomes (KEGG) pathway enrichment analyses by the R language. The former analysis, GO, as a commonly used and versatile bioinformatics tool, allows to identify gene functional annotations by biological process (BP), cellular component (CC) or molecular function (MF) categories, independently, while KEGG, also a frequently mentioned bioinformatics database, contains a large number of bioinformatics approaches and efficiently facilitates data analysis. Similarly, *P* < .01 was set as cutoff values.

### 2.3 Protein‐protein interaction (PPI) network construction, modular analysis and Hub genes identification

To analyse protein interactions, the PPI network was established with the help of the STRING online database (version 11.0; http://string-db.org/). The minimum required interaction score was set as medium confidence >0.4. The initial PPI network created with the online tool was to some extent complicated, so the Cytoscape software (version 3.7.2) was utilized to visualize and draw the interactions between proteins. In addition, the MCODE plug‐in in Cytoscape was adopted to explore the important modules in PPI network (the default parameters). The genes with top‐ten node degrees are defined as hub genes.

### 2.4 Verification of hub genes in intersection results

After identifying the intersection hub genes from three groups of data, the data of GSE150316 (https://www.ncbi.nlm.nih.gov/geo/query/acc.cgi?acc=GSE150316) obtained after bioinformatics analysis were used for verification. We found out the expression matrices of the hub genes corresponding to our research in this data set that contains enough samples of patients. And we imported the data of the infection group and the control group of them into GraphPad Prism (version 8.0.2) for *t* tests and non‐parametric tests. Finally, we choose *P* < .05 as the standard to screen the hub genes.

## 3 RESULTS

### 3.1 Identification of DEGs in infected SARS‐CoV‐2 cell lines

**Figure 1:**
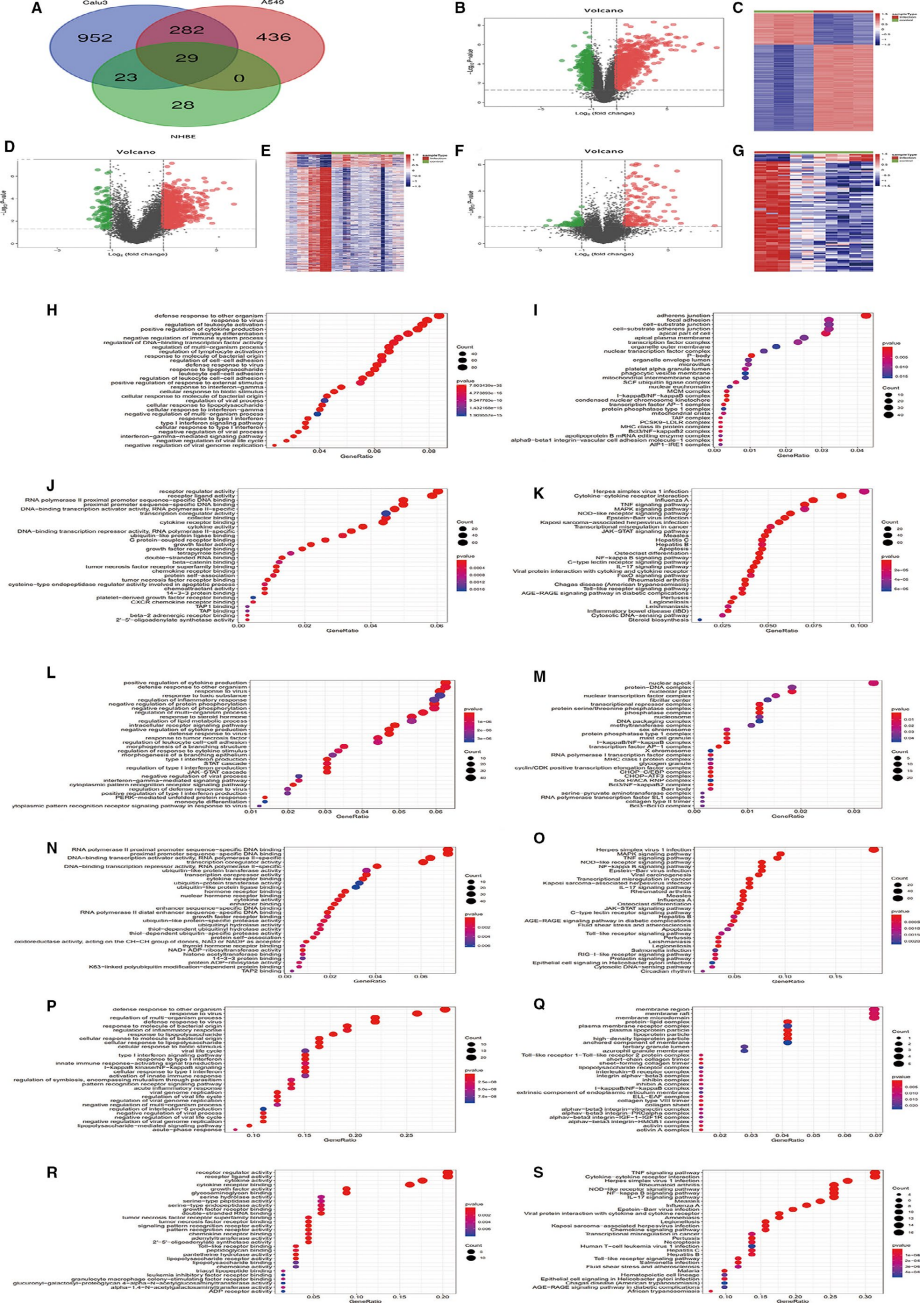
We identified 29 common DEGs from three sets of data (GSE147507). Different colour areas represent different data sets. Crossed regions indicate co‐expressed DEG. DEG was identified by classical *t* test, and the statistically significant DEG was defined as *P* < .01 and [logFC] > 1 as the screening criteria (A). At the same time, in Calu3 group, A549 group, and NHBE group, the volcano graphs of DEGs expression are all based on *P* < .01 and |logFC| > 1, black dots indicate genes with no significant difference, red dots indicate up‐regulated genes, green dots Represents the down‐regulated genes (B, D, F). The heat map of Calu3 group contains 3 SARS‐CoV‐2 infection samples and three control samples for DEGs expression (C), the heat map of A549 group contains 12 SARS‐CoV‐2 infection samples and 19 DEGs expression control samples (E), the heat map of the NHBE group contains three SARS‐CoV‐2 infection samples and seven control samples for DEGs expression (G). The GO annotation and KEGG pathway enrichment analysis of target genes in Calu‐3 group, A549 group and NHBE group are shown below. In the Calu‐3 group, (H) Enriched functional BP of the target genes; (I) Enriched CC of the target genes; (J) Enriched MF of the target genes; (K) Enriched KEGG pathways of the target genes. In the A549 group, (L) Enriched functional BP of the target genes; (M) Enriched CC of the target genes; (N) Enriched MF of the target genes; (O) Enriched KEGG pathways of the target genes. In the NHBE group, (P) Enriched functional BP of the target genes; (Q) Enriched CC of the target genes; (R) Enriched MF of the target genes; (S) Enriched KEGG pathways of the target genes

The original microarray data of GSE147507 related to SARS‐CoV‐2 were obtained at Gene Expression Omnibus (GEO). The data of GSE147507 were divided into three groups according to the differences of cell lines, namely Calu‐3, A549 and NHBE. With *P* < .01 and |logFC| > 1 as the screening criteria, a total of 1286, 747 and 80 DEGs were extracted from Calu‐3, A549 and NHBE groups, respectively (Table S1). In addition, the DEGs of the three groups were analysed through intersection, and finally 29 DEGs that were all continuously up‐regulated in the three groups were obtained (Figure 1A). Based on the data of GSE147507, three groups of volcano maps (Figure 1B,D,F) and heat maps (Figure 1C,E,G) were developed independently by R language, showing the significantly different distribution of each group.

### 3.2 GO function enrichment analysis of the DEGs

Composed of the biological pathway (BP), the CC, and the MF, GO enrichment analysis for the DEGs in three groups of Calu‐3, A549 and NHBE were shown in Table S2 and Figure 1H‐J,L‐N,P‐R.

### 3.3 KEGG pathway analysis

KEGG enrichment analysis was conducted on all DEGs and corresponding *P*‐value and *P*‐adjust values of each pathway were obtained. Subsequently, the top 10 channels of KEGG significance in each group were sorted out after the processing of a large amount of data. Taking more details into consideration, the gene names corresponding to each pathway were also marked in the Table S3. Dot plots were mapped for each set of data to give a more intuitive description of the results of KEGG analysis (Figure 1K,O,S).

The analysis of the results displayed that although the data derive from different types of samples, some pathways, such as TNF signalling pathway, NF‐kappa B signalling pathway, IL‐17 signalling pathway, NOD‐like receptor signalling pathway, and DEG, all showed upward trends.

### 3.4 PPI network analysis

**Figure 2:**
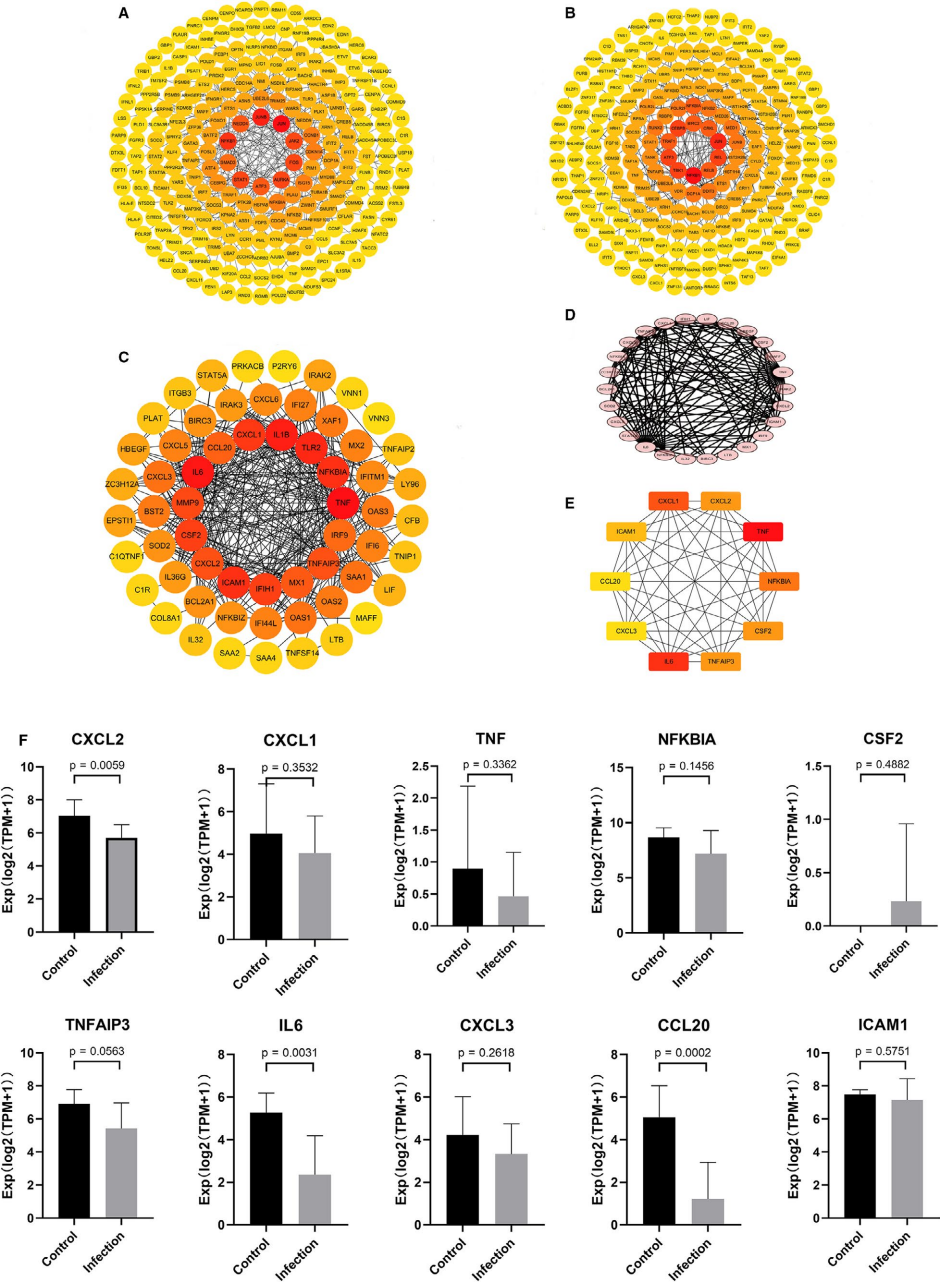
Construction of the PPI network and Verification of hub genes. (A) PPI network of Calu‐3 group. (B) PPI network of A549 group. (C) PPI network of NHBE group. (D) the intersected PPI network. (E) the hub genes of intersected PPI network. (F) Verification of hub genes

The STRING online tool was applied to construct PPI networks for the three groups independently, together with the intersected PPI network of DEGs data of the three groups for the sake of better understanding the interaction between proteins. It turned out that the PPI network of the Calu‐3 group has 234 nodes and 265 edges (Figure 2A), that of A549 group 220 nodes and 224 edges (Figure 2B), that of NHBE group 60 nodes and 364 edges (Figure 2C), and the intersected PPI network 29 nodes and 129 edges (Figure 2D). In addition, this study identified the hub genes with top‐ten node degrees in the intersected PPI network: CXCL1, CXCL2, TNF, NFKBIA, CSF2, TNFAIP3, IL6, CXCL3, CCL20 and ICAM1 (Figure 2E).

### 3.5 Verification of hub genes

In consideration of the rigorousness of this study, data from the GSE150316 gene data set were used to verify the 10 hub genes obtained. With *P* < .05 as the standard, it was found that only the analytic results of CXCL2, IL6 and CCL20 genes were statistically significant (Figure 2F).

## 4 DISCUSSION

This study obtained gene expression profiles of SARS‐CoV‐2 from GEO database and performed DEGs screening, GO and KEGG analysis, so as to understand the biological functions of these DEGs and report meaningful enrichment pathways. Subsequently, PPI analysis was conducted to identify the hub genes that play a key regulatory role in the pathologic process of infection.

Based on GO enrichment analyses of the DEGs among three groups, it was found that the response to virus, defence response to virus, and response to type I interferon all have high enrichment scores in the BP. These findings were with those from previously published studies which documented that the occurrence of coronavirus infection causes the body to initiate an innate immune response and trigger IFN gene up‐regulation to achieve the antiviral status.^6^ Besides, the CC category of Calu‐3 and A549 in enrichment analyses was I‐kappaB/NF‐kappaB complex and transcription factor AP‐1 complex. Transcription factors NF‐kappaB and AP‐1 make a big different in T cell activation processes.^7^ Interestingly, the CC is associated with high‐density lipoprotein particle in NHBE cell, implying that SARS‐CoV‐2 may regulate the lipid composition, lipid synthesis and signalling of host cell.^8^

According to KEGG analysis, after SARS‐CoV‐2 infection, there were four signalling pathways in the three groups changing jointly, among which the TNF signalling pathway transform is the most significant. This study analysed the significantly altered signalling pathways after SARS‐CoV‐2 infection, found out the possible pathogenic mechanisms and organisms of antiviral mechanisms, to provide new ideas for its treatment. In the NF‐kappa B signalling pathway, NF‐kB as a key transcription factor is crucial for innate and adaptive immunity. Studies have shown that the M protein of SARS‐CoV interacts with IKKb, inhibits the degradation of IkBa protein and the expression of NF‐kB‐dependent Cox‐2, so it is reasonable to believe that SARS‐CoV can evade immune responses by changing the gene expression of key inflammatory molecules.^9^ In TNF signalling pathway, Penicillium marneffei is a human pathogen that exists in macrophages and threatens immunocompromised patients. After infection with Penicillium marneffei, the body produces an important defence mechanism that induces TNF‐α production via extracellular signal‐regulated kinase (ERK) 1/2 to resist Pseudomonas marneffei.^10^ In addition, HCV‐infected cells will affect IFN‐α/β induction and response, which may inhibit IFN‐α/β induction by viral protease‐mediated cleavage of MAVS and TRIF, thereby inhibiting its antiviral effect against HCV.^11^ These studies indicate that the up‐regulation of TNF pathway may be beneficial for the inhibition of SARS‐CoV‐2.

After the above research and analysis, PPI networks were constructed by STRING, and from the intersection, 10 central genes were obtained, which were verified with data from GSE150316 database for the sake of rigorousness of scientific studies. The results showed that the analytic results of IL‐6, CXCL2 and ICAM‐1 were statistically significant, which suggested that these three genes possibly play a key regulatory role in the course of SARS‐CoV‐2 infection. And there are studies to back that up. For example, studies have discovered a significant increase in IL‐6 expression in patients with COVID‐19. In line with the principle that inhibited expression of IL‐6 can produce an obvious anti‐inflammatory effect,^12^ it is expected by some researchers that IL‐6 blockers be used to treat cytokine release syndrome caused by COVID‐19,^13^ thus saving patients’ lives. On the other hand, CXCL2 is also a cytokine highly expressed in infections cause by various viruses, such as Zika Virus,^14^ which will promote its expression and mediate an inflammatory response. ICAM‐1 was encoded by Group 2 innate lymphoid cells to reduce lung inflammation by destroying the homoeostasis and function of ILC2s.^15^ At the same time, the overexpression of ICAM‐1 and knockdown can also promote and block the production of rhinovirus, indicating that they also have certain regulatory effects on virus transfection.

In spite that this study included data from multi‐type samples, it had certain limitations. For one thing, the data studied is relatively small in size and may not be universal enough. For another, as the samples from which the data were extracted were mostly artificially cultured, this study lacked live samples, which would also compromise the reliability of this study.

## CONFLICT OF INTEREST

The authors declare that they have no competing interests.

## AUTHOR CONTRIBUTIONS

**Tian‐Ao Xie:** Data curation (equal); Project administration (equal); Writing‐original draft (lead); Writing‐review & editing (lead). **Meng‐Yi Han:** Data curation (equal); Writing‐original draft (equal); Writing‐review & editing (equal). **Xiao‐Rui Su:** Data curation (equal); Writing‐original draft (equal); Writing‐review & editing (equal). **Hou‐He Li:** Data curation (equal); Writing‐original draft (equal); Writing‐review & editing (equal). **Ji‐Chun Chen:** Data curation (equal); Writing‐original draft (supporting); Writing‐review & editing (equal). **Xuguang Guo:** Data curation (lead); Project administration (lead); Writing‐original draft (lead); Writing‐review & editing (lead).

## Supporting information

Table S1

Table S2

Table S3

## Data Availability

Not applicable.
