# Supplementary material for: Identification of Hub genes associated with infection of three lung cell lines by SARS‐CoV‐2 with integrated bioinformatics analysis
Source: J Cell Mol Med. 2020 Sep 14;24(20):12225–30. doi: 10.1111/jcmm.15862 (PMC7579704; doi:10.1111/jcmm.15862)
Supplement: Supplementary file 1 — Table S1 [file JCMM-24-12225-s001.docx]

Supplementary Table S1. The following table lists three groups of up-regulated and down-regulated differential genes after infection. It is worth noting that 29 differentially expressed genes (DEG) were identified in the intersection of the three groups of data, all of which are up-regulated genes.

| Groud | DEGs | Gene terms |
| --- | --- | --- |
| Calu3 | Up-regulated | PPP1R15A,CYP1A1,DUSP5,FUT3,IFIT3,PIM1,TRIM22,  RSAD2,IRF1,CXCL3,CTGF,CXCL2,CSF2,CH25H,SP110,KLF4,IL6,MX1,IKZF3,OASL,ZBP1,SLC25A37,NFKBIA,  FUT2,MAFF,CCL20,IL1B,KCNJ15,APOBEC3G,LAMP3,  PTX3,RND1,IL1A,G0S2,PTPRH,IFITM1,ICAM1,MX2,  OAS2,PLAU,IFIT1,UBE2L6,TNFAIP2,TAP1,IFNL2,  EPSTI1,CD274,ZNF385C,CSRNP1,ATF3,IFNL3,STAT2,  OPTN,USP18,DUSP1,SDC4,C15orf48,CYR61,GADD45A,  BATF2,DHX58,CXCL1,DUOX2,IFI35,CXCL10,TNFAIP3,  CCL5,PARP12,UBD,OAS1,TXNIP,TMEM140,SEPSECS,  TRIM21,NEAT1,EGOT,CX3CL1,TNF,H1F0,ZFP36,  SLC15A3,SAA4,C1S,ETS2,IFNB1,MB21D2,KDM6B,  GSDMB,DENND3,HBEGF,TRAF1,GBP5,RASGRP3,  RTP4,CFLAR,EGR2,KMO,PML,PLAT,IFIT2,NEURL3,  CXCL11,LAP3,IRF9,CCL2,IFIH1,GARS,ASNS,GJB4,  SERPINB9,STC2,TDRD7,KRTAP2-3,IFNL1,ATF4,KRT23,  XAF1,TNIP1,EDN1,DUOX1,ANKRD33B,HSH2D,  SEMA7A,TRANK1,APOL4,HES1,SERPINE1,ETV6,  CCL22,IFITM2,HLA-E,SNPH,GBP1,AMOTL2,GBP4,  PTGER4,SOCS3,TIPARP,TPKC,THEMIS2,TUFT1,INHBA,  FLT3LG,NR1D1,CTH,WARS,PDGFB,MAP3K8,IFI44L,  LGALS9,IRAK2,CARS,FGF19,TRIM38,KYNU,NUB1,  ZNFX1,ISG20,SP140L,APOBEC3F,SP100,PHF11,RBM11,  CYLD,ITGAM,BMP2,SAT1,C1QTNF1,HELZ2,IDO1,  SYNPO,GBP2,FSTL3,CSF3,HMGN2P46,EDN2,GATA6,  PARP10,PARP14,SLC1A4,ZC3HAV1,PTAFR,ARRDC3,  CASP1,NUAK2,LCN2,RNF19B,HCG4,BTN2A2,C6orf223,  GADD45B,TLK2,PSMB8,TRIB1,TBC1D22B,KLF6,  NLRC5,SYTL3,LST1,DDX60L,N4BP1,MYD88,HERC5,  IRF6,SOD2,PCK2,NLRP3,BBC3,ANKRD1,LIF,JARID2,  FOSB,NCOA7,SLC43A1,GEM,TNFSF10,APOL6,EGR1,  APOBEC3D,COX6B2,ISG15,ETS1,ANKLE2,PLAUR,  IFI44,HLA-F,ADRB2,FA2H,IL15RA,NLRP1,NEDD9,  KLF10,FOS,DTX3L,VCAM1,UBASH3A,YARS,FBXO6,  XBP1,IRF7,GTPBP2,IER3,TRIM25,TNFRSF10B,  BHLHE40,RGMB,CMPK2,DAB2IP,C3,FUT1,ALDH1L2,  HDX,SAMD9L,JUN,CNP,B3GNT7,PHACTR4,IFI16,  PLEKHA4,PRDM1,SERPINB7,F3,TRAFD1,CD22,DAPP1,  RARRES3,PLA2G6,NUAK1,ZC3H12C,VEGFA,IFIT5,  DUSP8,IRS2,CYP2B6,DDX58,PSPH,ASS1,CCNL1,  NDUFA4L2,EPB41L4A-AS1,NR4A2,DEPDC7,SYNGR3,  PER1,APOL2,KRT17,NFKB1,FILIP1L,DSG3,SERTAD2,  NCF2,PSORS1C1,WFDC5,GSE1,CHD2,PDGFA,  MARCH4,IL15,S100P,HRASLS2,NMI,PDE4B,EFNA1,  CXorf38,PPP2R2A,SMAD3,PNPT1,TTC39B,IL32,ADM2,  SLC7A5,TRIM5,DEFB4A,JDP2,TNFAIP6,CLK4,DCP1A,  CD69,SMAD7,FOXO1,RASGEF1B,AMIGO2,BACH2,  ETV7,FOSL1,RASGRP1,PARP9,SESN2,SPSB1,GMPR,  MT2A,C6orf58,NFKB2,IL12A,REC8,ATXN2L,TP53INP2,  PLA1A,BDNF,ZNF140,UBQLNL,C1R,CSF1,ATP2C2,  THBS1,GCKR,IRF2,LAMB3,MIR155HG,SRGN,PHLDB2,  RSRC2,DRAM1,CDC14A,SP8,TRIM16,GPT2,  CEACAM19,TSC22D2,RNF213,ARHGEF28,PMAIP1,  TRIM14,CITED2,OAS3,PLA2G4C,APOL3,GPCPD1,  SOCS2,MAST4,RBMS2,DUOXA2,FBXO32,ALS2CL,  TMPRSS3,CDKN1A,AKAP12,SERPINB1,BCL2L11,  ABTB2,NAV2,SLC5A1,ZNF442,DUSP16,NKX3-1,PCK1,  CREB5,KBTBD2,PLSCR1,SOX9,INHBE,ZNF185,  MAP1LC3B,GPR132,LMO2,SERPINB2,NFKBID,  LINC00494,ZSWIM4,TNFSF13B,UBA7,SMIM6,AJUBA,  LYN,TICAM1,ABL2,RAB42,RCAN1,GBP1P1,XRN1,  AMN1,PPP4R4,CMTM3,SAMD9,TMPRSS2,SSTR2,  SLFN5,GATA3,MOB3C,ULBP1,NFATC2,ZC3H4,NR4A3,  B4GALT5,BCAR3,CCDC174,CD55,ZC3H12A,SAMHD1,  MTHFD2,BDKRB2,DDX60,ADAR,TNFRSF11B,SEC16B,  HERC6,FLNB,CYTH4,ABCA12,SLC25A28,NR4A1,  SLC3A2,LTB,ZFAS1,TLR6,HIVEP2,KIF21B,TYMP,  CEBPG,TFAP2A,FOXO3,PSTPIP2,C6orf222,JUNB,  SLC1A3,CSRNP2,MXD1,DUSP10,ZNF184,ECE1,MIR614,  SPRY2,STAT1,DLC1,BTN3A1,SEMA3A,PTGS2,EPC1,  NFE2L3,MEIS1,SIK1,ZNF44,MIR21,PIM3,NEDD4,TLR1,  RIPK2,ACTN2,PRKD2,STK10,TLR3,IFNGR2,PNRC1,  PLEKHG7,IFITM3,ANKRD11,ERRFI1,LAMA2,ZCCHC2,  ST3GAL1,FOXK1,L1CAM,ZNF264,FST,ZBTB20,TRIM56,  C6orf132,BHLHE41,PSAT1,STAT5A,HIVEP1,PTK2B,  BCL9L,KLHDC7B,ZFP36L1,EIF2AK2,CD68,PSMB9,  TUBE1,CCR1,EHD4,PLD1,RREB1,S1PR2,HCG27,CLK1,  GPR37,FAM214A,RND3,PIP5K1A,SMURF1,RELB,  ZBTB10,OTUD1,PPP2R5B,SHROOM2,ZNF543,SULT1C2,  SECTM1,TAP2,ZBTB43,IFNL4,FGD6,DHRS9,JAK2,  FSD1L,BCL10,CEACAM5,PGLYRP2,ZCCHC8,POU2F3,  IFNGR1,COL16A1,TP53BP2,ARRDC4,ROCK1P1,GDF15,  ZNF274,SIRPB2,TGFB2,CYP1B1,OGFR,MUC1,BCL3,  SBNO2,SLC6A9,PA2G4P4,C18orf25,RIMKLB,FAP,  KIAA0040,PROSER2,GTPBP1,PEAR1,ZNF267,C5orf56,  KRTAP1-1,NFKBIE,PRSS3P2,ZNF419,MB,DDO,  CEACAM1,TNFRSF9,ZFP36L2,CALCOCO2,C19orf66,  EPHA2,BIRC3,NAV3,KDM6A,ST6GALNAC5,RASSF9,  SRFBP1,NGFR,RIN2,N4BP3,MAP3K13,SDS,GZF1,  POGZ,SLC25A25,CYP3A5,IFI6,KRT80,SAMSN1,CYP2J2,  PLK3,NPR1,SERPINA6,BCL6,DIAPH2,POU2F2,NPFFR2,  NFKBIZ,PPM1K,RAET1L,GBX2,ADM,ALDH1A3,  MAP1B,ZNF462,RUNX1,TUBB2B,ERN1,ZNF805,PSG4,  TMCC1,DYRK1A,LIPH,NR1I2,SUN3,MXRA5,FGF2,  BCL2A1,PARP8,ZFX,ZNF222,MMP13,CGB5,KRT6B,  TNFSF14,NR3C1,RAB11FIP1,HMOX1,CASP7,NUPR1,  CHAC1,LATS2,LIFR,ULK1,TNFRSF1B,BCL2L14,  C15orf39,ZNF441,VEGFC,ZNF217,IL22RA1,RFTN1,  DDX59,RBM43,ZNF484,ADAM8,ADPRM,RIOK3,  PLEKHG5,CD70,SERPINA10,SCN3A,USP42,TAGAP,  RPS6KA2,CDKN2AIP,NAPSB,MYOF,MCTP1,CREBRF,  CLEC7A,ATP13A4,NOD2,ARID5B,FOSL2,SYT1,HLA-J,  SAMD4A,AHRR,ARL4D,TNFSF15,HIF1A-AS2,ZNF791,  JAG1,NFE2L2,GLI2,CNOT4,KIAA1217,RICTOR,  STARD13,LRRN3,FFAR2,RNF144B,STX11,LHB,NCK1,  IL23A,NAMPT,NFAT5,SH2B3,GBP3,BEST3,COL9A2,  SOX4,RAB8B,CDKN2B,TMEM40,SSH1,HSD17B14,  SKIL,GIMAP2,GAB2,BST2,KRT16,ASPHD2,KCNV1,  UGCG,RAI14,PLD6,HAND1,NR1D2,VEZF1,LDLR,  KPNA7,AFAP1,ZNF14,FAM122C,PPP1R15B,TIAM2,  ETV3,KLF9,GFPT2,PPP4R1L,ZEB1,RASSF4,  RABGAP1L,SPEN,SGK1,PTGER2,ADAMTS9,ASAP1,  DCLRE1C,BPGM,PELI1,FAM84B,MSL2,EGLN3,EREG,  USP30-AS1,SLC34A2,DDX43,IL2RG,PCGF5,CARD16,  TANK,ZNF224,NRCAM,TRPS1,HDAC9,MPZ,TSPAN2,  CALD1,LRIF1,TRIM36,RPL13AP20,SLC25A51,CDK17,  ZNF697,CPEB3,ATF7IP,ZNF254,TAGLN,HRH1,HCP5,  PARD6B,TAF1D,CLUHP3,CXCL9,C8orf31,KLF11,  TREX1,ZNF564,PAWR,ANGPTL4,RNF19A,MACC1,  USP43,SHOX2,CAB39,PDP1,TJP1,UHRF1BP1,FRS2,  PIWIL4,BACH1,TRIO,PMS2CL,OTUD4,HIST1H1D,  HELB,MORC3,HSPB8,GOLT1A,B3GNT5,OGFRL1,  CXXC4,ARHGEF38,ZNF311,FAS,CSGALNACT1,  TNFRSF10A,GNB4,ANKRD12,DNAJB4,NCCRP1,ZNF83,  TMEM156,IL4I1,INTS6,CXCL5,CCL17,SH3TC1,CPEB2,  CLDN1,PTER,NUDCD1,RSF1,GUCA1B,TOB2P1,  PLXNA2,PAPOLG,ZBTB21,TRIM29,TCP11L2,AGO2,  ASAP2,ZNF277,SNAPC1,COLEC10,ODF3B,STK38L,CP,  BAZ1A,LATS1,SEC24A,RASSF6,RNF111,RBAK,ATXN7,  PDCD1LG2,SPTBN1,USP25,ZNF627,QKI,KIAA1551,  FAM160A1,PRSS1,CTSO,IL17C,FRMD6,SAA2,  LINC00853,CLEC4E,ZNF620,ACHE,CEACAM7,ARID4B,  PIK3AP1,PTPN12,SUSD3,ICAM4,RSL24D1,FLRT3,  CPXM1,MIER1,GCNT4,TET3,FNDC3B,STAT4,PER2,  KLHL29,SLC28A3,ZNF608,SIX4,RLF,MIR3191,ZNF562,  PICALM,ZNF460,ERCC4,LCA5,GNA13,ADORA2A,  ZBTB38,TCF7L2,ZNF292,HIST1H2BG,SNORA66,  SNORD94,ITSN2,RHCG,C10orf55,MALAT1,FRMD4B,  EGFR,STRN3,AFF4,PDE4D,BTG2,ZNF280D,MIR4645,  CPEB4,ODF3L1,VNN1,MIR146A,FEM1C,ZNF107,KLF5,  LINC00525,PATL2,BAZ2B,ARL5B,PDE10A,FAM83E,  SAMD8,HINT3,ZBTB33,SOCS1,FOXN2,PIK3R1,  SPRR2D,TNFAIP8,PPTC7,SWT1,USP53,IL18R1,CIITA,  PPP4R2,PRRG1,CCL28,ZNF675,TET2,JMJD1C,MIR4517,  ACTBL2,MAML2 |
|  | Down-regulated | NSDHL,HNRNPAB,VIL1,TM4SF4,KIFC1,ASB9,PAGR1,  SPAG5,FDFT1,DBI,NT5DC2,CYB561,MCM5,ASF1B,  SHMT1,NEFL,FEN1,PCYT2,PEBP1,SRSF2,DHCR7,  NME1,EMP2,CSPG5,FAM229B,GJB1,BDH1,NCAPD2,  PTDSS1,ACAT2,PHB,TYMS,NEB,UPK1B,FDPS,POP5,  SLIRP,STEAP3,TM7SF2,EBP,AIF1L,TMEM97,ARMC6,  LSS,ACSS2,IMPA2,ZWINT,C21orf58,CCNB1,KPNA2,  METTL7B,ALG1,FAM81A,LIG1,TMA7,HSPA1A,  METTL7A,ALDH16A1,IFT122,DCTPP1,DHCR24,TPX2,  MAOB,ANXA10,CLDN2,LMNB2,TUBA1B,THEM6,  CDCA3,HSPA8,MARC1,MVD,RAB36,NTHL1,EMP3,  SYT12,SNRNP25,TACC3,GALM,MANF,MANEAL,SGK2,  DLEU1,CENPM,PRPS1,TUBB4B,MPND,WDR54,MCM6,  DANCR,CENPA,RNASEH2C,BIRC5,TESC,FAM83D,  IMP3,MIR31HG,PCSK9,CASR,HOGA1,SAPCD2,  CDKN2C,TONSL,SAC3D1,FBXO9,JMJD8,H2AFX,  PXMP2,MMAB,SLC9A3R1,ANXA9,CENPO,LSM7,DDC,  WDR90,KIF18B,IL17RB,RARRES2,LMNB1,ADI1,  ZFYVE21,ANKRD54,DECR2,COX8A,OST4,MLEC,  COMMD9,NDUFB2,POLR2F,CCNF,EPN3,SPC24,  RECQL4,COMMD4,PMVK,NDUFS3,PFN1,NUDC,  GTSE1,KIF20A,SOSTDC1,SNCA,MVK,MPV17L,CDKN3,  ROGDI,NCLN,UCP2,UROD,POLD1,SLC39A4,WWOX,  MXD3,PRDX2,SAMD1,CYS1,TBCD,LRRC45,DNAAF3,  NPBWR1,CD320,TPPP3,RRM2,KCNK2,PLK1,THOP1,  TMEM147,CRYM,DERL3,AURKA,DTYMK,ANXA13,  IGFBP6,CHRNA5,ATP6V0E2,FASN,RERG,SLC25A10,  FGFR3,FADS2,POLD2,AMDHD2,C1orf53,CDC45,SCGN,  HR,CACYBP,LRRC6,FBXW9,TSTA3,PGP,SPP1,  TCTEX1D2,TUSC2,DGCR6L,SCARA3,GINS2,SLC2A8,  FZD1,RIBC2,DNLZ,TNFAIP8L1,ATP6V0D1,DCXR,  SPC25,CTXN1,CCDC34,C7orf50,MRPL12,DPP3,GCAT,  PRR15L,NDUFB11,ID1,SIGMAR1,SF3B5,AGFG2,  WDR34,IGSF9,DSEL,ZNF580,CETN2,TMEM121,CAT,  RAMP1,ANKS4B,CNFN,SQLE,HPDL,MDH1B,SIVA1,  MME,PSRC1,BCL7C,SDF2L1,CAPS,TRNP1,C1orf122,  NDUFB7,CENPV,PIN1,QPCTL,SAP30,FAM173B,TBL3,  UQCR11,TMEM129,NHP2,SCD,POLE4,KIF12,CRIP2,  AADAT,BCL2L12,KCNMB4,DPCD,CHDH,MCM4,  SLC22A18AS,MPC2,IHH,PACSIN3,AGMAT,ARHGEF19,  POLR3K,TMEM213,VWA1,C9orf116,PPP1R16A,  TIMM8B,PRADC1,TRIM54,MKI67,GAMT,LAGE3,  SLC27A3,MIF,CHST7,AQP1,AURKAIP1,ENDOG,  MFSD3,CDCA2,B9D1,MALL,IGFLR1,CDKN2A,F12,  SMAD6,SARS2,HSPBP1,MRPL41,ADCK5,FAM173A,  VAV3,PIF1,PDGFD,BAD,CAPN8,SSNA1,ZNF511,NUBP2,  BLOC1S1,PARD6A,FDXR,NME3,PCOLCE2,PLA2G10,  RASL11A,RPS10,HES6,SKP2,DLGAP5,POLR2L,ZNF771,  MNS1,OIP5,ROMO1,HPGD,ZNF488,MEDAG,GCSH,  ENKD1,GLTPD2,TOP2A,RAB26,CCDC96,CTNND2,  HSPE1,CHTF18,MRPS26,C2CD4B,LRRC26,MACROD1,  CENPI,HTRA1,CLEC11A,AGR3,TPGS1,TGFBR3L,  TSPAN12,ALKBH7,NOC4L |
| A549 | Up-regulated | RELB,STX11,RASGRP1,EREG,SOD2,IL23A,N4BP3,  MAFF,IL1A,ZNF799,SLC6A15,IRAK2,DRAM1,KLF9,  ZNF641,PRX,ZNF615,ZNF184,ZNF711,ZFP37,TRAF1,  SNHG1,DDX60,ZNF805,IRF9,BCL2A1,IER3,CXCL2,  TNFAIP3,PLEKHF2,BRAF,ZC3H12C,SPHK1,CXCL3,  TRIM36,EFNA1,PHLDA1,THAP9-AS1,IRF1,PRKCE,  ZNF567,TNFAIP8,LIF,ZNF34,STAT1,CSF1,REL,PPP1R18,  SOCS2,ZNF627,DDIT4,IL6,RBPJ,BIRC3,SDC4,HIVEP1,  NFKB1,SELE,NFKBIZ,TMEM156,PTX3,ZNF274,ZNF776,  FOXD1,PARP9,ZFAND1,SNX16,B3GALNT2,NFKBIA,  ZSCAN12,ICAM5,CHIC2,CDC14A,FOXC2,NECAP1,  NUAK2,CCNB1IP1,PARP12,PPP4R1L,HBP1,HCFC2,  ZNF614,DTX3L,KLF11,ZNF430,RAB33B,SKIL,HIVEP2,  TIPARP,FOXO1,IL20RB,ZNF211,MYSM1,ZNF639,HLA-F,  JUN,KLF10,ZNF280B,NFKBIE,FST,ZBTB10,ZNF518B,  RASA2,PPP1R15A,NOC3L,TLK2,ZNF845,TRANK1,  UBE2L6,ZNF555,ARID3B,INTS6,ELOVL7,RNF169,  NKX3-1,DNAH17,PLEKHG2,USPL1,DDIT3,WTAP,  PCF11,PRDM1,CEP95,IFRD1,MDM4,CSF3,TNF,ZBTB6,  TAF1A,UFM1,NFKB2,SIX4,NFIL3,RASSF8,TAF1D,  USP12,TRIM25,ZNF222,AEBP2,ARRDC4,ZNF14,  KDM6B,NIPAL4,MITD1,RBM39,SNAP25,UTP15,  MB21D2,FLCN,COLEC11,ICAM1,ZNF134,FGF18,HSF2,  SOCS6,ZNF764,PLD6,SENP7,PID1,FOXN2,CREBRF,  ABL2,IKZF5,MYNN,SAMD4A,ZNF451,ZNF77,CHD2,  PDE4B,RNF19B,NPHS1,MSL2,DNAJC27,C11orf96,GPR3,  HES7,CYLD,ODF3B,ZFP36L1,EPB41L4A-AS1,BCL3,  ODC1,GPBP1L1,C5orf51,ZKSCAN8,ZNF436,ZNF383,  TBC1D12,CLK1,SMURF2,CEBPB,CHST2,ZNF484,  ZBTB21,BHLHE41,EPC1,MAP1LC3B,NBPF10,KLF6,  KIAA2026,MARCH7,SNORD65,ELL2,CCL20,PPP1R15B,  CCNT1,SNORA48,ZNF830,CLUHP3,ETS1,ZC3H12A,  MARCKS,CEP85L,VDR,GADD45B,DMTF1,THUMPD2,  RHOU,ZNF12,BTG1,COL2A1,ZEB2,PPM1E,MORC3,  DUSP1,SOCS3,NR1D1,SERTAD2,IRF7,THAP1,ZMYM5,  KLF7,DDX58,RIMKLB,CCNL1,CCDC174,NEURL3,  PNRC1,SPRED1,EFNA3,CNOT4,CLK4,GATA6,PCDH1,  HLA-B,VEGFA,VEZF1,ZNF432,YAF2,PIGA,LYSMD3,  CDCP1,TGM2,ZBTB2,LAT2,DDX60L,NCOA7,BIRC2,  MED1,LYST,FNIP1,ARMCX3,ARL5B,CRY1,  CSGALNACT2,H1F0,ZNF57,SNIP1,MCTP1,ZNF558,  KCTD11,B3GNT5,BMP2,CSF2,LTB,GPBP1,PIM1,JMY,  FRS2,CRKL,ZBED5,CCL2,GRHL1,C3orf38,RCHY1,  WDR26,ZBTB43,ANKRD49,MEX3C,ZNF461,SAMD9L,  MAP3K8,ZNF8,HIST1H2BG,ADNP2,ZBTB5,SRFBP1,  ZBTB34,GEM,XRN1,ITSN2,GAS5,PPTC7,NXT2,TANK,  ZEB1-AS1,RUNX2,PAPOLG,APOL6,DCAF4L1,IFI16,  USP43,ZNF669,ZC3H11A,GABPB1,ARRDC3,USP53,  TBC1D22B,PER1,SAT1,ESF1,TET3,NFKBID,SNORA8,  ZCCHC10,RLF,ZNF460,HIF1A-AS2,GPATCH8,MIER1,  TGS1,CCDC81,CA2,PPP4R4,SMNDC1,NR4A3,IFIT5,  CD200R1,ARMCX5,ZNF24,TAB2,HRH1,ZNF23,SOCS5,  ZNF317,GBP3,SLC7A2,DCP1A,CDK17,PPP1R3B,  ZNF121,SAMD9,NRBF2,SDE2,FOXP1,PARP10,CDKN1B,  CHD1,UBE2B,JUNB,SNORA47,THBD,SCAF8,PARP14,  RSF1,PRPF39,ZNF543,ARHGAP40,CEP350,CXCL1,  NBEAP1,RB1CC1,NAV3,STMN4,THAP2,CXCL5,ZNFX1,  ETV3,KDM5B,HELZ2,TNFRSF9,MED13,BTN2A1,  FCHO2,TBK1,CFLAR-AS1,TANC2,ADM2,CCNT2,  USP37,SAA2,RBAK,ZBTB1,SNORD58A,EAF1,RSRC2,  TET2,CYP1B1,ARID4B,IFIH1,LIG4,C1S,ZNF292,  ZBTB11,RNASEK,ADPRM,DBP,CCNG2,CSRNP1,C1D,  ANKRD12,SNORA25,DUSP16,TAB3,RAI14,NMD3,  SNORA1,PDE10A,UGCG,MXI1,GADD45A,ZNF160,  PPP4R2,RPL23AP53,PER3,CDKL3,EXOC8,UBASH3A,  PRKXP1,SNORD59A,MIER3,TSC22D2,CDK5R1,  BHLHE40,IER5,PTGS2,KIAA0895,CETN4P,INO80D,  RLIM,YTHDC1,STK38L,UBR5,STRN3,RBM48,NR1D2,  PLSCR1,DUSP8,MCL1,PMAIP1,PPIL4,MMP10,RIOK3,  SCARNA15,RFPL3S,DCUN1D3,LAMTOR3,SOCS1,  REC8,C2orf66,MBIP,ZNF721,ZNF267,TAP1,ZBTB33,  HIST1H2BM,SMCHD1,RNMT,PPM1K,C1R,MIR590,  CD300LB,PNRC2,BCL10,OLR1,C1orf52,IFIT3,GNA13,  HERC5,DUSP5,ANKRD50,PDP1,TMEM158,CYP1A1,  RNF6,ANGPTL4,DLX2,TAF7,JMJD1C,CLEC4A,  LACC1,ZSCAN12P1,RSBN1,VAMP2,SETX,FOSL1,  ZNF778,RRAGC,NCK1,PHLDB2,RBBP6,WAC,BLZF1,  CD83,RND3,ABTB2,NIPBL,NFE2L2,ZNF131,FGD6,  HIST1H4I,HSPA13,HIST2H2BE,LPAR6,HDAC9,SCUBE2,  ATF3,IGFBP1,LINC00641,CCL4,SPRY1,LTN1,GBP1,  STAT2,ZNF143,TOPORS,AREG,GREM1,ATP6V1G2,  SNORA28,HBEGF,SP110,SNORA80B,ZNF674-AS1,  OTUD1,MIR221,USP15,C3,RYBP,EGR2,GOLGA6C,  ZNF79,EEA1,CDKN2AIP,ZNF770,SPAG9,ZNF217,  CTDSPL2,SNORD6,MAPK6,ZNF92,BTG3,MX1,AKAP12,  TAF13,CPEB4,CHD9,TXNIP,COL4A3BP,VTRNA1-1,  C3orf52,BBC3,STC2,PTP4A1,SNORA12,CREB5,GPR142,  BTAF1,MXD1,PURB,MIR561,FEM1B,WEE1,GPCPD1,  ADAM20P1,IL32,SNORA31,HIST2H2BC,FRMD6,  RNF219,ACBD3,LINC00472,SNORA81,FEM1C,STAT5A,  ZFC3H1,CFL1P1,EPM2AIP1,SNORD18B,NUFIP2,  C3orf35,SUMO4,PPP1R10,NRIP1,IFI44,RICTOR,LBP,  TAOK1,KDM6A,TVP23B,ZNF254,BACH1,TOP1P1,  MIR3685,ZCCHC3,IFIT2,RSPRY1,ANKRD33B,  RNF138P1,CMPK2,SNORA63,ARC,SNORD20,LRIF1,  RIT1,MAP4K5,SCYL2,MIR3064,ASS1,ITGA2,FAM126B,  SNORA27,ERRFI1,HIST1H2AK,ADM,MAP4K3,ELF1,  MIR5047,WSB1,LAMP3,ZNF281,SNORA62,KLKP1,  NEAT1,NSRP1,HIST1H1D,HIST3H2BB,CLIC4,OASL,  THEMIS2,PLIN2,BMPER,ZNF596,CD274,SNORD21,  SNORD100,SLC30A1,RNF11,ZNF644,TNS1,FILIP1L,  SNORD46,TNFAIP6,CLEC2B,MIR7-1,ACHE,PNN,  ZNF654,RNU6ATAC,GLIPR1,ZRANB2,SOCS4,IER2,  BDP1,LINC00115,MED26,RANBP6 |
|  | Down-regulated | EPHX1,H19,TBXAS1,SLC22A18AS,SYNGR4,NT5DC2,  CIDEC,TUBB4A,DEGS2,RAP1GAP,GPX2,PHB,  SLC9A3R1,POLR2F,PTMS,AKR1B10,G6PD,POLR2L,  NDUFA2,DHCR24,AP1M2,FADS2,AGR2,FCGBP,BCAS1,  ALDH3A1,TECR,SERPINA6,THEM6,KRT4,ATP6V0E2,  SLC25A10,AMBP,EBP,NDUFB7,TM7SF2,CNFN,DHCR7,  RAB26,CYBA,DHRS4L2,HABP2,PROC,FGFR3,WDR34,  FGFBP1,METTL7B,NUBP2,MVK,HSPBP1,UPK3B,  NRGN,ISOC2,APOH,MVD,CDH16,TM4SF4,BRICD5,  ZDHHC8P1,HLA-DMB,FGFR4,SNRNP25,SCARA5,  ABHD14A,CLDN2,ARL2,NDUFS7,KHK,TK1,LGALS2,  FASN,SPDEF,CPLX2,RECQL4,MCM5,DDC,TPPP3,  SHMT1,C10orf91,PRODH2 |
| NHBE | Up-regulated | IRAK2,PGLYRP4,HBEGF,C1QTNF1,IL36G,CCL20,  ZC3H12A,TNIP1,HMGN2P46,VNN3,SOD2,CES1P1,  CXCL3,INHBA,BCL2A1,OAS1,IFI27,XAF1,MAFF,OAS2,  TNFAIP3,MX1,IRF9,VNN1,MX2,CXCL1,IL6,IFITM1,  BPGM,TNFAIP2,HEPHL1,IL1B,LTB,TNF,TNFSF14,SAA2,  MED4-AS1,IFI44L,CXCL5,MMP9,SAA4,OAS3,PLAT,IFI6,  C15orf48,P2RY6,BIRC3,SAA1,STAT5A,CXCL2,BST2,  TRIML2,EPSTI1,CFB,IL32,CSF2,ICAM1,COL8A1,DTX2,  TLR2,NFKBIZ,C1R,PDZK1IP1,IFIH1,NFKBIA,DRAM1,  PLA2G4C,MIR5194,MIR601,CXCL6,ITGB3,  SNORD116-14,IRAK3,MIR103A2,LIF |
|  | Down-regulated | EXTL2,EAF2,CCDC138,LY96,PRKACB |
| Intersection | Up-regulated | CXCL3,C1R,SOD2,HBEGF,LIF,STAT5A,ZC3H12A,TNF,  LTB,NFKBIZ,CCL20,DRAM1,TNFAIP3,IL6,BCL2A1,  IRAK2,CXCL2,ICAM1,CXCL1,MX1,IL32,CXCL5,IFIH1,NFKBIA,SAA2,IRF9,MAFF,CSF2,BIRC3 |
|  | Down-regulated | / |
