# Supplementary material for: Identification of Hub genes associated with infection of three lung cell lines by SARS‐CoV‐2 with integrated bioinformatics analysis
Source: J Cell Mol Med. 2020 Sep 14;24(20):12225–30. doi: 10.1111/jcmm.15862 (PMC7579704; doi:10.1111/jcmm.15862)
Supplement: Supplementary file 2 — Table S2 [file JCMM-24-12225-s002.docx]

Supplementary Table S2. GO analyses results of differentially expressed genes (top 5 according to P-value).

| Group | Category | ID | Description | Count | P-value | GeneID |
| --- | --- | --- | --- | --- | --- | --- |
| Calu-3 | BP | GO:0009615 | Response to virus | 91 | 7.90344E-35 | CYP1A1/IFIT3/TRIM22/RSAD2/IRF1/IL6/MX1/OASL/IL1B/APOBEC3G/IFITM1/MX2/OAS2/IFIT1/IFNL2/IFNL3/STAT2/DHX58/DUOX2/CXCL10/TNFAIP3/CCL5/OAS1/TNF/IFNB1/RTP4/PML/IFIT2/IRF9/IFIH1/IFNL1/CCL22/IFITM2/GBP1/IFI44L/LGALS9/TRIM38/ISG20/APOBEC3F/ZC3HAV1/LCN2/NLRC5/HERC5/NLRP3/APOBEC3D/ISG15/IFI44/DTX3L/IRF7/TRIM25/IFI16/IFIT5/DDX58/IL15/TRIM5/FOSL1/PARP9/IL12A/IRF2/PMAIP1/OAS3/BCL2L11/PLSCR1/TICAM1/GATA3/ZC3H12A/SAMHD1/DDX60/ADAR/STAT1/TLR3/IFNGR2/IFITM3/TRIM56/EIF2AK2/IFNL4/IFNGR1/BCL3/BIRC3/IFI6/RIOK3/IL23A/GBP3/BST2/POLR3K/CXCL9/TREX1/BAD/SKP2/HTRA1/MIR146A |
|  |  | GO:0051607 | Defense response to virus | 70 | 3.85579E-29 | TRIM22/RSAD2/IRF1/IL6/MX1/OASL/IL1B/APOBEC3G/MX2/OAS2/IFIT1/IFNL2/IFNL3/STAT2/DHX58/CXCL10/TNFAIP3/OAS1/IFNB1/RTP4/PML/IRF9/IFIH1/IFNL1/GBP1/IFI44L/TRIM38/ISG20/APOBEC3F/ZC3HAV1/NLRC5/HERC5/NLRP3/APOBEC3D/ISG15/DTX3L/IRF7/TRIM25/IFI16/IFIT5/DDX58/IL15/TRIM5/PARP9/IRF2/PMAIP1/OAS3/PLSCR1/TICAM1/ZC3H12A/SAMHD1/DDX60/ADAR/STAT1/TLR3/IFITM3/TRIM56/EIF2AK2/IFNL4/BIRC3/IFI6/RIOK3/IL23A/GBP3/BST2/POLR3K/CXCL9/TREX1/SKP2/HTRA1 |
|  |  | GO:0034340 | Response to type I interferon | 41 | 2.79693E-24 | IFIT3/RSAD2/IRF1/MX1/OASL/IFITM1/MX2/OAS2/IFIT1/STAT2/USP18/IFI35/OAS1/IFNB1/IFIT2/IRF9/XAF1/IFITM2/HLA-E/ISG20/SP100/GBP2/PSMB8/NLRC5/MYD88/IRF6/EGR1/ISG15/HLA-F/IRF7/IRF2/OAS3/SAMHD1/ADAR/STAT1/MIR21/IFITM3/TRIM56/IFI6/BST2/TREX1 |
|  |  | GO:0034341 | Response to interferon-gamma | 55 | 2.80171E-24 | TRIM22/IRF1/OASL/IFITM1/ICAM1/OAS2/CCL5/UBD/OAS1/TRIM21/GBP5/PML/IRF9/CCL2/EDN1/IFITM2/HLA-E/GBP1/GBP4/SOCS3/LGALS9/TRIM38/KYNU/NUB1/SP100/GBP2/PARP14/PTAFR/CASP1/NLRC5/IRF6/HLA-F/VCAM1/IRF7/TRIM25/ASS1/NMI/TRIM5/PARP9/MT2A/IRF2/OAS3/FLNB/SNCA/STAT1/TLR3/IFNGR2/IFITM3/JAK2/IFNGR1/CALCOCO2/BST2/CLDN1/SOCS1/CIITA |
|  |  | GO:0060337 | Type I interferon signaling pathway | 40 | 4.34223E-24 | IFIT3/RSAD2/IRF1/MX1/OASL/IFITM1/MX2/OAS2/IFIT1/STAT2/USP18/IFI35/OAS1/IFNB1/IFIT2/IRF9/XAF1/IFITM2/HLA-E/ISG20/SP100/GBP2/PSMB8/NLRC5/MYD88/IRF6/EGR1/ISG15/HLA-F/IRF7/IRF2/OAS3/SAMHD1/ADAR/STAT1/MIR21/IFITM3/IFI6/BST2/TREX1 |
|  | CC | GO:0033256 | I-kappaB/NF-kappaB complex | 4 | 1.46013E-05 | NFKBIA/NFKB1/NFKB2/BCL3 |
|  |  | GO:0000932 | P-body | 12 | 0.000421115 | APOBEC3G/TRIM21/ZFP36/APOBEC3F/APOBEC3D/TRIM5/DCP1A/AJUBA/XRN1/ZC3H12A/ZFP36L1/AGO2 |
|  |  | GO:0000778 | Condensed nuclear chromosome kinetochore | 4 | 0.000439202 | CCNB1/REC8/CENPA/PLK1 |
|  |  | GO:0005912 | Adherens junction | 49 | 0.000825983 | RND1/ICAM1/PLAU/SDC4/LAP3/AIF1L/PLAUR/NEDD9/HSPA1A/CDCA3/HSPA8/SMAD7/IRF2/PHLDB2/AKAP12/ZNF185/AJUBA/LYN/ZFYVE21/FLNB/PFN1/DLC1/ACTN2/TBCD/L1CAM/PTK2B/RND3/PIP5K1A/SHROOM2/JAK2/FGFR3/FAP/FZD1/CEACAM1/EPHA2/CAT/MME/JAG1/AFAP1/KLF11/TJP1/FRS2/TRIM29/RPS10/PTPN12/FLRT3/GNA13/FRMD4B/EGFR |
|  |  | GO:0035976 | Transcription factor AP-1 complex | 3 | 0.002151347 | FOS/JUN/JUNB |
|  | MF | GO:0005125 | Cytokine activity | 43 | 4.16687E-16 | CXCL3/CSF2/IL6/IL1B/IL1A/IFNL2/IFNL3/CXCL10/CCL5/CX3CL1/TNF/IFNB1/CXCL11/IFNL1/EDN1/INHBA/FLT3LG/CSF3/LIF/TNFSF10/VEGFA/IL15/IL32/IL12A/CSF1/TNFSF13B/CMTM3/TNFRSF11B/LTB/SECTM1/IFNL4/TGFB2/SPP1/FGF2/TNFSF14/CD70/TNFSF15/IL23A/NAMPT/CXCL9/MIF/IL17C/CCL28 |
|  |  | GO:0005126 | Cytokine receptor binding | 50 | 2.08912E-15 | CXCL3/CSF2/IL6/CCL20/IL1B/IL1A/CXCL10/CCL5/CX3CL1/TNF/IFNB1/TRAF1/CFLAR/CXCL11/CCL2/IFNL1/MYD88/LIF/TNFSF10/YARS/DAB2IP/VEGFA/IL15/SMAD3/DEFB4A/SMAD7/IL12A/CSF1/SOCS2/TNFSF13B/GATA3/LTB/STAT1/JAK2/TGFB2/TNFSF14/LIFR/SIVA1/VEGFC/CD70/TNFSF15/IL23A/SH2B3/CXCL9/MIF/FRS2/SMAD6/CCL17/PIK3R1/CCL28 |
|  |  | GO:0048018 | Receptor ligand activity | 67 | 2.40083E-12 | CXCL3/CSF2/IL6/IL1B/IL1A/IFNL2/IFNL3/CXCL1/CXCL10/CCL5/CX3CL1/TNF/IFNB1/HBEGF/CXCL11/STC2/IFNL1/EDN1/INHBA/FLT3LG/PDGFB/FGF19/CSPG5/BMP2/CSF3/EDN2/LIF/TNFSF10/VEGFA/PDGFA/IL15/IL32/ADM2/MANF/IL12A/CSF1/INHBE/TNFSF13B/CMTM3/TNFRSF11B/LTB/TYMP/SEMA3A/CD320/SECTM1/IFNL4/GDF15/TGFB2/SPP1/ADM/FGF2/TNFSF14/VEGFC/CD70/TNFSF15/JAG1/IL23A/NAMPT/CXCL9/MIF/MACC1/PDGFD/COLEC10/IL17C/FLRT3/CLEC11A/CCL28 |
|  |  | GO:0030545 | Receptor regulator activity | 69 | 7.16941E-12 | CXCL3/CSF2/IL6/IL1B/IL1A/IFNL2/IFNL3/CXCL1/CXCL10/CCL5/CX3CL1/TNF/IFNB1/HBEGF/CXCL11/STC2/IFNL1/EDN1/INHBA/FLT3LG/PDGFB/FGF19/CSPG5/BMP2/CSF3/EDN2/LIF/TNFSF10/VEGFA/PDGFA/IL15/IL32/ADM2/MANF/IL12A/CSF1/PCSK9/INHBE/TNFSF13B/CMTM3/TNFRSF11B/LTB/TYMP/SEMA3A/FST/CD320/SECTM1/IFNL4/GDF15/TGFB2/SPP1/ADM/FGF2/TNFSF14/VEGFC/CD70/TNFSF15/JAG1/IL23A/NAMPT/CXCL9/MIF/MACC1/PDGFD/COLEC10/IL17C/FLRT3/CLEC11A/CCL28 |
|  |  | GO:0001227 | DNA-binding transcription repressor activity, RNA polymerase II-specific | 39 | 4.02778E-08 | ATF3/ETS2/HES1/ETV6/NR1D1/JARID2/BHLHE40/IFI16/PRDM1/NFKB1/JDP2/FOXO1/BACH2/ETV7/ZNF140/GATA3/NFATC2/TFAP2A/FOXO3/MXD1/NFE2L3/MXD3/FOXK1/ZBTB20/BHLHE41/HIVEP1/GZF1/ZNF217/ARID5B/SKIL/HAND1/NR1D2/ETV3/ZEB1/ZNF224/TRPS1/BACH1/HES6/BTG2 |
| A549 | BP | GO:0009615 | Response to virus | 40 | 1.44942E-11 | IL23A/DDX60/IRF9/TNFAIP3/IRF1/STAT1/DDIT4/IL6/BIRC3/PARP9/DTX3L/TNF/TRIM25/BCL3/ODC1/ZC3H12A/IRF7/DDX58/BIRC2/LYST/IFI16/IFIT5/GBP3/TBK1/IFIH1/PLSCR1/PMAIP1/RIOK3/IFIT3/HERC5/CYP1A1/FOSL1/CCL4/GBP1/STAT2/MX1/IFI44/ZCCHC3/IFIT2/OASL |
|  |  | GO:0051607 | Defense response to virus | 31 | 4.01297E-10 | IL23A/DDX60/IRF9/TNFAIP3/IRF1/STAT1/DDIT4/IL6/BIRC3/PARP9/DTX3L/TRIM25/ZC3H12A/IRF7/DDX58/BIRC2/LYST/IFI16/IFIT5/GBP3/TBK1/IFIH1/PLSCR1/PMAIP1/RIOK3/HERC5/GBP1/STAT2/MX1/ZCCHC3/OASL |
|  |  | GO:0030522 | Intracellular receptor signaling pathway | 32 | 5.86873E-09 | IRAK2/DDX60/TNFAIP3/BIRC3/NFKBIA/PHB/NKX3-1/UFM1/CYLD/VDR/NR1D1/IRF7/DDX58/BIRC2/MED1/CRY1/PIM1/CRKL/PER1/NR4A3/TAB2/FOXP1/IFIH1/TAB3/UBR5/STRN3/NR1D2/RIOK3/RNF6/TAF7/NRIP1/ZCCHC3 |
|  |  | GO:0032479 | Regulation of type I interferon production | 20 | 2.02384E-08 | RELB/TNFAIP3/IRF1/STAT1/REL/NFKB1/UBE2L6/POLR2F/NFKB2/TRIM25/POLR2L/CYLD/IRF7/DDX58/IFI16/TBK1/IFIH1/RIOK3/HERC5/ZCCHC3 |
|  |  | GO:0036499 | PERK-mediated unfolded protein response | 9 | 2.193E-08 | PPP1R15A/DDIT3/AGR2/PPP1R15B/CCL2/NCK1/NFE2L2/ATF3/IGFBP1 |
|  | CC | GO:0033256 | I-kappaB/NF-kappaB complex | 4 | 1.48983E-06 | NFKB1/NFKBIA/NFKB2/BCL3 |
|  |  | GO:0008287 | Protein serine/threonine phosphatase complex | 8 | 4.17354E-05 | PPP1R15A/PPP1R15B/PPP4R4/PPP1R3B/PPP4R2/IER5/NCK1/PPP1R10 |
|  |  | GO:1903293 | Phosphatase complex | 8 | 4.17354E-05 | PPP1R15A/PPP1R15B/PPP4R4/PPP1R3B/PPP4R2/IER5/NCK1/PPP1R10 |
|  |  | GO:0000164 | Protein phosphatase type 1 complex | 4 | 0.000163052 | PPP1R15A/PPP1R15B/PPP1R3B/NCK1 |
|  |  | GO:0035976 | Transcription factor AP-1 complex | 3 | 0.000405347 | JUN/DDIT3/JUNB |
|  | MF | GO:0000978 | RNA polymerase II proximal promoter sequence-specific DNA binding | 46 | 2.1672E-10 | IRF9/IRF1/STAT1/RBPJ/FOXD1/FOXC2/SKIL/ZNF639/JUN/KLF10/ARID3B/NKX3-1/DDIT3/PRDM1/NFKB2/SIX4/NFIL3/AEBP2/CHD2/CEBPB/BHLHE41/KLF6/ETS1/VDR/NR1D1/IRF7/THAP1/KLF7/ZBTB2/MED1/CRY1/GRHL1/RUNX2/IFI16/PER1/NR4A3/BHLHE40/NR1D2/FOSL1/ZNF143/EGR2/ZNF217/MXD1/NRIP1/ELF1/ZNF281 |
|  |  | GO:0000987 | Proximal promoter sequence-specific DNA binding | 46 | 9.58404E-10 | IRF9/IRF1/STAT1/RBPJ/FOXD1/FOXC2/SKIL/ZNF639/JUN/KLF10/ARID3B/NKX3-1/DDIT3/PRDM1/NFKB2/SIX4/NFIL3/AEBP2/CHD2/CEBPB/BHLHE41/KLF6/ETS1/VDR/NR1D1/IRF7/THAP1/KLF7/ZBTB2/MED1/CRY1/GRHL1/RUNX2/IFI16/PER1/NR4A3/BHLHE40/NR1D2/FOSL1/ZNF143/EGR2/ZNF217/MXD1/NRIP1/ELF1/ZNF281 |
|  |  | GO:0001228 | DNA-binding transcription activator activity, RNA polymerase II-specific | 41 | 3.11846E-09 | MAFF/IRF1/REL/RBPJ/NFKB1/FOXD1/FOXC2/ZNF639/JUN/KLF10/DDIT3/NFKB2/SIX4/HSF2/CREBRF/CEBPB/KLF6/ETS1/DMTF1/ZEB2/KLF7/VEZF1/GRHL1/RUNX2/NR4A3/ZNF24/JUNB/ZNF292/DBP/CSRNP1/PLSCR1/DLX2/FOSL1/NFE2L2/ZNF131/ATF3/ZNF143/EGR2/BACH1/ELF1/IER2 |
|  |  | GO:0001227 | DNA-binding transcription repressor activity, RNA polymerase II-specific | 26 | 3.68374E-07 | HIVEP1/NFKB1/SKIL/FOXO1/PRDM1/NFIL3/AEBP2/IKZF5/CEBPB/BHLHE41/ZEB2/NR1D1/THAP1/ZBTB2/ZBTB5/IFI16/ETV3/MXI1/BHLHE40/NR1D2/ATF3/ZNF217/MXD1/BACH1/SPDEF/ZNF281 |
|  |  | GO:0003714 | Transcription corepressor activity | 23 | 3.10243E-06 | RELB/ZNF274/PARP9/DDIT3/NFIL3/AEBP2/ZNF451/BHLHE41/NR1D1/YAF2/MED1/JUNB/KDM5B/C1D/MXI1/BHLHE40/RLIM/HDAC9/ATF3/RYBP/MXD1/NRIP1/ZNF281 |
| NHBE | BP | GO:0009615 | Response to virus | 19 | 2.84815E-17 | ZC3H12A/OAS1/IFI27/OAS2/TNFAIP3/MX1/IRF9/MX2/IL6/IFITM1/IL1B/TNF/IFI44L/OAS3/IFI6/BIRC3/BST2/IFIH1/IRAK3 |
|  |  | GO:0098542 | Defense response to other organism | 21 | 1.97444E-16 | PGLYRP4/CCL20/ZC3H12A/OAS1/IFI27/OAS2/TNFAIP3/MX1/IRF9/MX2/IL6/IL1B/TNF/IFI44L/OAS3/IFI6/BIRC3/BST2/TLR2/IFIH1/CXCL6 |
|  |  | GO:0051607 | Defense response to virus | 16 | 9.54012E-16 | ZC3H12A/OAS1/IFI27/OAS2/TNFAIP3/MX1/IRF9/MX2/IL6/IL1B/IFI44L/OAS3/IFI6/BIRC3/BST2/IFIH1 |
|  |  | GO:0060337 | Type I interferon signaling pathway | 11 | 8.57819E-14 | OAS1/IFI27/XAF1/OAS2/MX1/IRF9/MX2/IFITM1/OAS3/IFI6/BST2 |
|  |  | GO:0071357 | Cellular response to type I interferon | 11 | 8.57819E-14 | OAS1/IFI27/XAF1/OAS2/MX1/IRF9/MX2/IFITM1/OAS3/IFI6/BST2 |
|  | CC | GO:0034364 | High-density lipoprotein particle | 3 | 0.000133988 | SAA2/SAA4/SAA1 |
|  |  | GO:0034358 | Plasma lipoprotein particle | 3 | 0.000388429 | SAA2/SAA4/SAA1 |
|  |  | GO:1990777 | Lipoprotein particle | 3 | 0.000388429 | SAA2/SAA4/SAA1 |
|  |  | GO:0032994 | Protein-lipid complex | 3 | 0.00045435 | SAA2/SAA4/SAA1 |
|  |  | GO:0005591 | Collagen type VIII trimer | 1 | 0.003854802 | COL8A1 |
|  | MF | GO:0005125 | Cytokine activity | 12 | 9.51941E-13 | IL36G/CXCL3/INHBA/IL6/IL1B/LTB/TNF/TNFSF14/IL32/CSF2/CXCL6/LIF |
|  |  | GO:0048018 | Receptor ligand activity | 14 | 4.58272E-10 | HBEGF/IL36G/CXCL3/INHBA/CXCL1/IL6/IL1B/LTB/TNF/TNFSF14/IL32/CSF2/CXCL6/LIF |
|  |  | GO:0005126 | Cytokine receptor binding | 11 | 6.63626E-10 | CCL20/CXCL3/IL6/IL1B/LTB/TNF/TNFSF14/CSF2/CXCL6/ITGB3/LIF |
|  |  | GO:0030545 | Receptor regulator activity | 14 | 1.11023E-09 | HBEGF/IL36G/CXCL3/INHBA/CXCL1/IL6/IL1B/LTB/TNF/TNFSF14/IL32/CSF2/CXCL6/LIF |
|  |  | GO:0001730 | 2'-5'-oligoadenylate synthetase activity | 3 | 6.15511E-08 | OAS1/OAS2/OAS3 |

Abbreviations: BP, biological process; CC, cellular component; GO, Gene Ontology; MF, molecular function.
