# Supplementary material for: Identification of Hub genes associated with infection of three lung cell lines by SARS‐CoV‐2 with integrated bioinformatics analysis
Source: J Cell Mol Med. 2020 Sep 14;24(20):12225–30. doi: 10.1111/jcmm.15862 (PMC7579704; doi:10.1111/jcmm.15862)
Supplement: Supplementary file 3 — Table S3 [file JCMM-24-12225-s003.docx]

Supplementary Table S3. The top ten significant pathways in KEGG analysis of each groups.

| Group | ID | Description | Count | P-value | P.adjust | GeneID |
| --- | --- | --- | --- | --- | --- | --- |
| Calu-3 | hsa04668 | TNF signaling pathway | 45 | 6.00268E-21 | 1.81881E-18 | IRF1/CXCL3/CXCL2/CSF2/IL6/NFKBIA/CCL20/IL1B/ICAM1/CXCL1/CXCL10/TNFAIP3/CCL5/CX3CL1/TNF/IFNB1/TRAF1/CFLAR/CCL2/ATF4/EDN1/SOCS3/MAP3K8/LIF/FOS/VCAM1/DAB2IP/JUN/NFKB1/IL15/CSF1/CREB5/JUNB/PTGS2/BCL3/BIRC3/CASP7/TNFRSF1B/VEGFC/NOD2/JAG1/FAS/CXCL5/PIK3R1/IL18R1 |
|  | hsa05164 | Influenza A | 48 | 4.85168E-15 | 7.3503E-13 | RSAD2/IL6/MX1/NFKBIA/IL1B/IL1A/ICAM1/OAS2/STAT2/CXCL10/CCL5/OAS1/TNF/IFNB1/PML/IRF9/CCL2/IFIH1/SOCS3/CASP1/FDPS/MYD88/NLRP3/TNFSF10/IRF7/TRIM25/TNFRSF10B/KPNA2/DDX58/NFKB1/IL12A/OAS3/TICAM1/TMPRSS2/ADAR/STAT1/TLR3/IFNGR2/EIF2AK2/JAK2/IFNGR1/CALCOCO2/KPNA7/FAS/TNFRSF10A/PRSS1/PIK3R1/CIITA |
|  | hsa04060 | Cytokine-cytokine receptor interaction | 58 | 1.15732E-10 | 1.1689E-08 | CXCL3/CXCL2/CSF2/IL6/CCL20/IL1B/IL1A/IFNL2/IFNL3/CXCL1/CXCL10/CCL5/CX3CL1/TNF/IFNB1/CXCL11/CCL2/IFNL1/CCL22/INHBA/BMP2/CSF3/LIF/TNFSF10/IL15RA/TNFRSF10B/IL15/IL32/IL12A/CSF1/INHBE/TNFSF13B/IL17RB/TNFRSF11B/LTB/IFNGR2/CCR1/IFNGR1/GDF15/TGFB2/TNFRSF9/NGFR/TNFSF14/LIFR/TNFRSF1B/IL22RA1/CD70/TNFSF15/IL23A/IL2RG/CXCL9/FAS/TNFRSF10A/CXCL5/CCL17/IL17C/IL18R1/CCL28 |
|  | hsa04064 | NF-kappa B signaling pathway | 29 | 1.17469E-09 | 8.89831E-08 | CXCL3/CXCL2/NFKBIA/IL1B/ICAM1/PLAU/CXCL1/TNFAIP3/TNF/TRAF1/CFLAR/CYLD/GADD45B/MYD88/VCAM1/TRIM25/DDX58/NFKB1/NFKB2/TNFSF13B/LYN/TICAM1/LTB/PTGS2/RELB/BCL10/BIRC3/BCL2A1/TNFSF14 |
|  | hsa04621 | NOD-like receptor signaling pathway | 40 | 2.0547E-09 | 1.24515E-07 | CXCL3/CXCL2/IL6/NFKBIA/IL1B/OAS2/STAT2/CXCL1/TNFAIP3/CCL5/OAS1/TXNIP/TNF/IFNB1/GBP5/IRF9/CCL2/GBP1/GBP4/GBP2/CASP1/MYD88/NLRP3/NLRP1/IRF7/JUN/IFI16/NFKB1/DEFB4A/OAS3/CASR/TICAM1/STAT1/RIPK2/BIRC3/NOD2/NAMPT/GBP3/CARD16/TANK |
|  | hsa04657 | IL-17 signaling pathway | 27 | 2.66275E-09 | 1.34469E-07 | CXCL3/CXCL2/CSF2/IL6/NFKBIA/CCL20/IL1B/CXCL1/CXCL10/TNFAIP3/TNF/CCL2/CSF3/LCN2/FOSB/FOS/JUN/NFKB1/DEFB4A/FOSL1/IL17RB/PTGS2/MMP13/CXCL5/CCL17/USP25/IL17C |
|  | hsa04625 | C-type lectin receptor signaling pathway | 28 | 8.72868E-09 | 3.77827E-07 | IRF1/IL6/NFKBIA/IL1B/STAT2/TNF/EGR2/IRF9/CCL22/CYLD/CASP1/NLRP3/JUN/NFKB1/NFKB2/IL12A/NFATC2/STAT1/PTGS2/RELB/BCL10/BCL3/PLK3/CLEC7A/IL23A/CCL17/CLEC4E/PIK3R1 |
|  | hsa05134 | Legionellosis | 19 | 3.81845E-08 | 1.44624E-06 | CXCL3/CXCL2/IL6/NFKBIA/IL1B/CXCL1/TNF/ITGAM/CASP1/MYD88/C3/HSPA1A/NFKB1/HSPA8/CLK4/NFKB2/IL12A/CLK1/CASP7 |
|  | hsa04061 | Viral protein interaction with cytokine and cytokine receptor | 26 | 6.49303E-08 | 2.18599E-06 | CXCL3/CXCL2/IL6/CCL20/CXCL1/CXCL10/CCL5/CX3CL1/TNF/CXCL11/CCL2/CCL22/TNFSF10/TNFRSF10B/CSF1/CCR1/TNFSF14/TNFRSF1B/IL22RA1/IL2RG/CXCL9/TNFRSF10A/CXCL5/CCL17/IL18R1/CCL28 |
|  | hsa05162 | Measles | 31 | 1.39678E-07 | 4.23224E-06 | IL6/MX1/NFKBIA/IL1B/IL1A/OAS2/STAT2/TNFAIP3/OAS1/IFNB1/IRF9/IFIH1/MYD88/BBC3/FOS/IRF7/JUN/HSPA1A/DDX58/NFKB1/HSPA8/IL12A/OAS3/ADAR/STAT1/STAT5A/EIF2AK2/IL2RG/FAS/BAD/PIK3R1 |
| A549 | hsa04668 | TNF signaling pathway | 30 | 5.6253E-17 | 1.45695E-14 | TRAF1/CXCL2/TNFAIP3/CXCL3/IRF1/LIF/CSF1/IL6/BIRC3/NFKB1/SELE/NFKBIA/JUN/TNF/ICAM1/BCL3/CEBPB/CCL20/SOCS3/BIRC2/CSF2/CCL2/MAP3K8/TAB2/JUNB/CXCL1/CXCL5/TAB3/PTGS2/CREB5 |
|  | hsa05168 | Herpes simplex virus 1 infection | 61 | 2.21529E-15 | 2.8688E-13 | ZNF799/ZNF641/ZNF615/ZNF184/ZFP37/IRF9/ZNF567/ZNF34/STAT1/ZNF627/IL6/BIRC3/NFKB1/NFKBIA/HCFC2/ZNF614/ZNF430/ZNF211/HLA-F/ZNF845/ZNF555/TNF/ZNF222/ZNF14/ZNF764/ZNF77/ZNF436/ZNF383/ZNF484/ZNF12/SOCS3/IRF7/DDX58/HLA-B/ZNF432/BIRC2/ZNF57/ZNF558/CCL2/ZNF461/ZNF8/ZNF669/ZNF460/TAB2/ZNF23/ZNF317/ZNF543/TBK1/RBAK/IFIH1/ZNF160/ZNF721/ZNF267/TAP1/ZNF778/STAT2/C3/ZNF79/HLA-DMB/ZNF254/ZNF596 |
|  | hsa04064 | NF-kappa B signaling pathway | 25 | 2.29332E-13 | 1.9799E-11 | RELB/TRAF1/BCL2A1/CXCL2/TNFAIP3/CXCL3/BIRC3/NFKB1/NFKBIA/TNF/NFKB2/TRIM25/ICAM1/CYLD/GADD45B/DDX58/BIRC2/LTB/TAB2/CXCL1/TAB3/PTGS2/BCL10/CCL4/LBP |
|  | hsa04657 | IL-17 signaling pathway | 21 | 1.07977E-10 | 6.99148E-09 | CXCL2/TNFAIP3/CXCL3/IL6/NFKB1/NFKBIA/JUN/CSF3/TNF/CEBPB/CCL20/CSF2/CCL2/TAB2/CXCL1/CXCL5/TBK1/TAB3/PTGS2/FOSL1/MAPK6 |
|  | hsa05323 | Rheumatoid arthritis | 19 | 5.14768E-09 | 2.6665E-07 | IL23A/IL1A/CXCL2/CXCL3/CSF1/IL6/JUN/TNF/ICAM1/CCL20/VEGFA/CSF2/LTB/CCL2/ATP6V0E2/CXCL1/CXCL5/ATP6V1G2/HLA-DMB |
|  | hsa04621 | NOD-like receptor signaling pathway | 25 | 6.16269E-08 | 2.66023E-06 | IRF9/CXCL2/TNFAIP3/CXCL3/STAT1/IL6/BIRC3/NFKB1/NFKBIA/JUN/TNF/IRF7/BIRC2/CCL2/TANK/IFI16/TAB2/GBP3/CYBA/CXCL1/TBK1/TAB3/GBP1/STAT2/TXNIP |
|  | hsa05169 | Epstein-Barr virus infection | 25 | 6.67393E-07 | 2.46935E-05 | RELB/IRF9/TNFAIP3/STAT1/IL6/RBPJ/NFKB1/NFKBIA/HLA-F/JUN/NFKBIE/TNF/NFKB2/ICAM1/GADD45B/IRF7/DDX58/HLA-B/TAB2/CDKN1B/TBK1/GADD45A/TAP1/STAT2/HLA-DMB |
|  | hsa04625 | C-type lectin receptor signaling pathway | 17 | 9.88906E-07 | 3.20158E-05 | RELB/IL23A/IRF9/IRF1/STAT1/IL6/NFKB1/NFKBIA/JUN/TNF/NFKB2/CYLD/BCL3/PTGS2/BCL10/STAT2/EGR2 |
|  | hsa04010 | MAPK signaling pathway | 31 | 1.29919E-06 | 3.73878E-05 | RELB/RASGRP1/EREG/IL1A/BRAF/EFNA1/CSF1/NFKB1/JUN/RASA2/DDIT3/TNF/NFKB2/FGF18/GADD45B/DUSP1/EFNA3/VEGFA/CRKL/MAP3K8/TAB2/FGFR3/DUSP16/GADD45A/DUSP8/LAMTOR3/DUSP5/AREG/FGFR4/TAOK1/MAP4K3 |
|  | hsa05203 | Viral carcinogenesis | 24 | 2.35398E-06 | 6.0968E-05 | TRAF1/IRF9/REL/RBPJ/NFKB1/NFKBIA/HLA-F/JUN/RASA2/NFKB2/IRF7/HLA-B/HIST1H2BG/CDKN1B/PMAIP1/HIST1H2BM/HIST1H4I/HIST2H2BE/HDAC9/C3/EGR2/CREB5/STAT5A/HIST3H2BB |
| NHBE | hsa04668 | TNF signaling pathway | 16 | 4.34258E-18 | 7.81664E-16 | CCL20/CXCL3/TNFAIP3/CXCL1/IL6/IL1B/TNF/CXCL5/MMP9/BIRC3/CXCL2/CSF2/ICAM1/NFKBIA/CXCL6/LIF |
|  | hsa04657 | IL-17 signaling pathway | 13 | 1.19196E-14 | 7.15176E-13 | CCL20/CXCL3/TNFAIP3/CXCL1/IL6/IL1B/TNF/CXCL5/MMP9/CXCL2/CSF2/NFKBIA/CXCL6 |
|  | hsa05323 | Rheumatoid arthritis | 13 | 1.19196E-14 | 7.15176E-13 | CCL20/CXCL3/CXCL1/IL6/IL1B/LTB/TNF/CXCL5/CXCL2/CSF2/ICAM1/TLR2/CXCL6 |
|  | hsa04064 | NF-kappa B signaling pathway | 13 | 4.12354E-14 | 1.85559E-12 | CXCL3/BCL2A1/TNFAIP3/CXCL1/IL1B/LTB/TNF/TNFSF14/BIRC3/CXCL2/ICAM1/LY96/NFKBIA |
|  | hsa04060 | Cytokine-cytokine receptor interaction | 16 | 2.05837E-11 | 7.41014E-10 | IL36G/CCL20/CXCL3/INHBA/CXCL1/IL6/IL1B/LTB/TNF/TNFSF14/CXCL5/CXCL2/IL32/CSF2/CXCL6/LIF |
|  | hsa05162 | Measles | 12 | 4.52877E-11 | 1.35863E-09 | OAS1/OAS2/TNFAIP3/MX1/IRF9/IL6/IL1B/OAS3/STAT5A/TLR2/IFIH1/NFKBIA |
|  | hsa04621 | NOD-like receptor signaling pathway | 13 | 5.76072E-11 | 1.48133E-09 | CXCL3/OAS1/OAS2/TNFAIP3/IRF9/CXCL1/IL6/IL1B/TNF/OAS3/BIRC3/CXCL2/NFKBIA |
|  | hsa05134 | Legionellosis | 8 | 1.94075E-09 | 4.36669E-08 | CXCL3/CXCL1/IL6/IL1B/TNF/CXCL2/TLR2/NFKBIA |
|  | hsa05164 | Influenza A | 11 | 7.64933E-09 | 1.52987E-07 | OAS1/OAS2/MX1/IRF9/IL6/IL1B/TNF/OAS3/ICAM1/IFIH1/NFKBIA |
|  | hsa04061 | Viral protein interaction with cytokine and cytokine receptor | 9 | 1.15552E-08 | 2.07993E-07 | CCL20/CXCL3/CXCL1/IL6/TNF/TNFSF14/CXCL5/CXCL2/CXCL6 |
